# Fatal Falls Overboard in Commercial Fishing — United States, 2000–2016

**DOI:** 10.15585/mmwr.mm6716a2

**Published:** 2018-04-27

**Authors:** Samantha L. Case, Jennifer M. Lincoln, Devin L. Lucas

**Affiliations:** 1 Western States Division, National Institute for Occupational Safety and Health, CDC.

Commercial fishing is one of the most dangerous jobs in the United States, with a 2016 work-related fatality rate (86.0 deaths per 100,000 full-time equivalent workers) 23 times higher than that for all U.S. workers (3.6) (*1*). Sinking vessels cause the most fatalities in the industry; however, falling from a fishing vessel is a serious hazard responsible for the second highest number of commercial fishing–associated fatalities (*2*,*3*). CDC’s National Institute for Occupational Safety and Health (NIOSH) analyzed data on unintentional fatal falls overboard in the U.S. commercial fishing industry to identify gaps in the use of primary, secondary, and tertiary prevention strategies. During 2000–2016, a total of 204 commercial fishermen died after unintentionally falling overboard. The majority of falls (121; 59.3%) were not witnessed, and 108 (89.3%) of these victims were not found. Among 83 witnessed falls overboard, 56 rescue attempts were made; 22 victims were recovered but were not successfully resuscitated. The circumstances, rescue attempts, and limited use of lifesaving and recovery equipment indicate that efforts to reduce these preventable fatalities are needed during pre-event, event, and post-event sequences of falls overboard. Vessel owners could consider strategies to prevent future fatalities, including lifeline tethers, line management, personal flotation devices (PFDs), man-overboard alarms, recovery devices, and rescue training.

A case of commercial fishing–associated overboard fall fatality was defined as a fatal traumatic injury resulting from an unintentional fall from a commercial fishing vessel in United States waters during 2000–2016. Fishermen often live on their vessels when working and are exposed to hazards while off duty; therefore, victims were considered to be at work for the entire time they were at sea. Cases were identified from NIOSH’s Commercial Fishing Incident Database, a national surveillance system that collects detailed information on all work-related fatalities in the fishing industry; data sources include U.S. Coast Guard investigative reports, local law enforcement reports, medical examiner documents, and news media. Records for each fall overboard were reviewed to determine the circumstances of the fall, including time in water, any use of survival or rescue equipment, recovery attempts, and administration of medical treatment. A descriptive analysis of event and decedent characteristics, including year, geographic region, fishery,* victim demographics, worker activity, primary cause of the fall, and contributing factors, was conducted. The trend in the number of fatal falls overboard over the course of the study period was evaluated using Poisson regression.

During 2000–2016, unintentional falls overboard resulted in 204 fatalities, representing 27.0% of all work-related deaths in the industry. Fall-overboard fatalities ranged from a high of 20 in 2003 to a low of five in 2016 (Figure 1). On average, the number of falls overboard decreased by 3.9% annually (incidence rate ratio = 0.961; p = 0.006).

**FIGURE 1:**
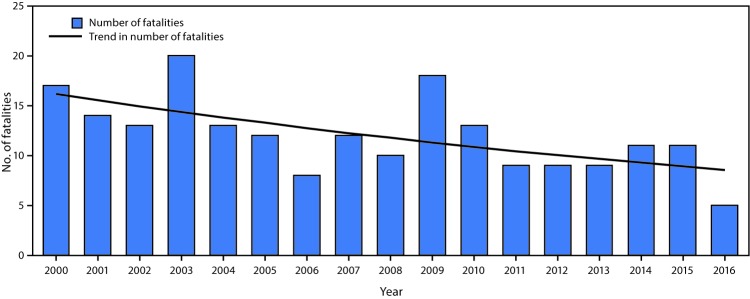
Number and trend* of unintentional fatal falls overboard (N = 204) in the commercial fishing industry, by year — United States, 2000–2016 * Significant decrease in the number of fatalities during 2000–2016 (Poisson regression, no exposure; incidence rate ratio = 0.961, p = 0.006).

Fatalities occurred most frequently on the East Coast (62; 30.4%), followed by the Gulf of Mexico (60; 29.4%), Alaska (51; 25.0%), and the West Coast (26; 12.8%). Five deaths occurred off the Hawaiian Coast. The Gulf of Mexico shrimp fishery had the highest number of fall-overboard deaths (34; 16.7%), followed by East Coast lobster (18; 8.8%), Alaska salmon drift gillnet (16; 7.8%), and East Coast scallop (10; 4.9%).

Among 187 (91.7%) decedents with information available on age, the median age was 43 years (range = 16–77 years). Overall, 202 (99.0%) decedents were male (Table). The majority of victims were employed as deckhands (120; 58.8%), and among 94 (46.1%) with information on years of experience, victims had a median of 16 years of experience in the fishing industry (range = 0–65 years). Nine victims (4.4%) were confirmed to have taken formal marine safety training.

**TABLE:** Characteristics of 204 unintentional fatal falls overboard in the commercial fishing industry — United States, 2000–2016

Characteristic (no. [%] known)	No. (% of known)
**Age group, yrs (187 [91.7])**	
≤24	17 (9.1)
25–44	84 (44.9)
45–64	79 (42.2)
≥65	7 (3.7)
Unknown (% of total)	17 (8.3)
**Gender (204 [100.0])**	
Male	202 (99.0)
Female	2 (1.0)
**Race/Ethnicity (144 [70.6])**	
Non-Hispanic	
White	72 (50.0)
Asian	29 (20.1)
American Indian/Alaska Native	16 (11.1)
Black/African American	8 (5.6)
Other	3 (2.1)
Hispanic	16 (11.1)
Unknown (% of total)	60 (29.4)
**Position (204 [100.0])**	
Operator	79 (38.7)
Deckhand	120 (58.8)
Other	5 (2.5)
**Experience, yrs (94 [46.1])**	
≤1	11 (11.7)
2–5	14 (14.9)
6–10	14 (14.9)
11–20	28 (29.8)
≥21	27 (28.7)
Unknown (% of total)	110 (53.9)
**Worker activity before fall (152 [74.5])**	
Traffic onboard	11 (7.2)
On watch	11 (7.2)
Working with fishing gear	
Preparing gear	10 (6.6)
Setting gear	35 (23.0)
Hauling gear	20 (13.2)
Handling gear on deck	12 (7.9)
Working with the catch	7 (4.6)
Off duty	34 (22.4)
Other	12 (7.9)
Unknown (% of total)	52 (25.5)
**Cause of fall (149 [73.0])**	
Lost balance	48 (32.2)
Trip/Slip	47 (31.5)
Gear entanglement	31 (20.8)
Struck by gear/object	14 (9.4)
Washed overboard	9 (6.0)
Unknown (% of total)	55 (27.0)

Among 152 (74.5%) fatalities for which information on victim activity preceding the fall was available, half (77; 50.7%) occurred while the victims were working with fishing gear, including setting gear (35; 23.0%), hauling gear onboard (20; 13.2%), and handling gear on deck (12; 7.9%). Falls also occurred while crewmembers were on deck while off duty (34; 22.4%). Among 149 (73.0%) cases where the cause of the fall was known, the leading causes were losing balance (48; 32.2%), tripping or slipping (47; 31.5%), and becoming entangled in gear (31; 20.8%). Of all 204 falls, the most commonly identified contributing factors included working alone (99; 48.5%), alcohol and drug use (37; 18.1%), and inclement weather (24; 11.8%).

The majority of falls (121; 59.3%) were unwitnessed, and most of these victims (108; 89.3%) were not located within an hour of the fall (Figure 2). For the 83 witnessed falls overboard, 56 (67.5%) rescue attempts were made, with 22 victims recovered but none successfully resuscitated.

**FIGURE 2:**
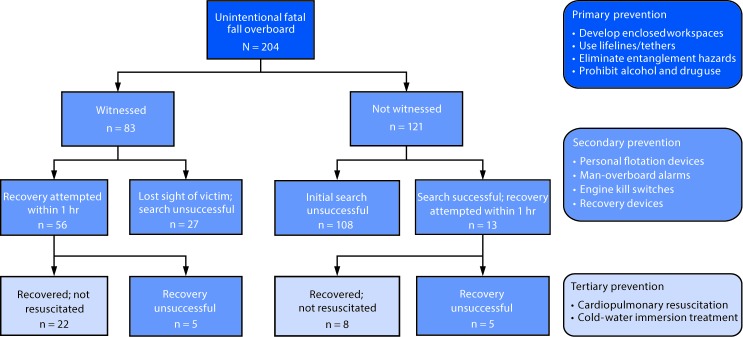
Recovery status of unintentional fatal fall overboard victims (N = 204) and associated prevention strategies — United States, 2000–2016

In all instances, none of the victims was wearing a PFD at the time of death. Among 19 (9.3%) events in which use of a life ring^†^ was noted, recovery attempts failed in most cases (14; 73.7%). A man-overboard alarm was only reportedly used in one event. Among the 30 crewmembers who were recovered from the water within an hour, cardiopulmonary resuscitation (CPR) was attempted on 21 (70.0%), but none could be resuscitated.

## Discussion

Preventing falls overboard is a priority area in fishing safety (*2*–*4*). Primary prevention strategies include creating enclosed workspaces, raising the gunnels^§^ on the vessel, and using lifelines and tethers where possible; vessel modifications should be conducted in consultation with a naval architect or engineer. Because of differences in fishing methods, workers in some fisheries are more exposed to entanglement hazards than are others, especially those who work with lines while setting gear (e.g., East Coast lobstermen). Engineering controls, such as line bins that catch excess line while hauling gear, can control hazards by reducing the amount of line on deck. In addition, enforcing drug- and alcohol-free policies on vessels might reduce the likelihood of crewmembers unintentionally falling from a vessel.

Without flotation, victims can drown within minutes after immersion in cold water through cold-shock responses, including hyperventilation and aspiration, as well as the deterioration of muscle function from lowered temperature, impeding swim efforts (*5*). Although federal regulations^¶^ mandate that commercial fishing vessels carry a PFD for each crewmember, there are no requirements for fishermen to wear them while working. Lack of PFD use is associated with workers’ negative perceptions and attitudes toward PFDs. Many fishermen recognize the effectiveness of PFDs to prevent drownings, but concerns regarding discomfort, cost, work interference, and potential for entanglement hinder widespread adoption throughout the industry (*6*,*7*). In 2008, NIOSH conducted a study in which participants in several Alaskan fisheries wore and evaluated various PFD types. Although preferences differed by fishery, each identified favorable PFDs that were acceptable to work in (*8*). On the basis of this research, one manufacturer worked collaboratively with the fishing industry and developed an innovative PFD that was responsive to workers’ concerns (*9*). Additional PFD evaluations have been conducted in the Pacific Northwest, Gulf of Mexico, and New England. Attempts to increase PFD use should continue, particularly given the increased commercial availability of comfortable and workable PFDs.

The majority of fatal falls overboard in this study were not observed. An unwitnessed fall overboard results in search and rescue delays and reduces the chances of a successful recovery. A man-overboard alarm is a small device worn by a worker that, in the event of water immersion, relays a signal to a receiver on the vessel and sounds an alarm to enable prompt rescue efforts. Use of this technology has not been widely adopted by the fishing industry despite its potential to save lives and be incorporated into work gear.

Although rescue attempts were made within 1 hour for 69 victims, over half (39; 56.5%) were unable to be recovered from the water, underscoring the difficulty of retrieving an overboard fall victim. Effective recovery devices, such as lifting slings, can provide additional flotation and help hoist the victim onto the vessel. Participation in marine safety training and drills can prepare crewmembers in man-overboard response and recovery. For fishermen who work alone, a reboarding ladder should be available on the vessel for self-rescue. Some man-overboard alarms include engine shutoff features that would keep the vessel nearby to facilitate reboarding.

None of the 30 crewmembers who were recovered onboard within 1 hour could be resuscitated. Successful treatment might be more likely if professional medical assistance were possible, a challenge when operating in remote locations. Having first-aid trained crewmembers administer CPR, prevent further heat loss, and rewarm the victim is a priority (*5*).

The findings in this report are subject to at least three limitations. First, the level of missing data varied among cases, and for at least one variable (years of experience), exceeded 50%. This circumstance might have introduced bias by underestimating certain fall or decedent characteristics when variables with missing data were analyzed. Second, denominator data were unavailable to enable calculation of fatality rates. A decreasing trend in the number of falls overboard was observed, but it is unclear if risk similarly declined. Finally, data were not available on nonfatal falls overboard. Comparison of fatal and nonfatal events might help identify factors associated with the successful rescue of crewmembers from the water.

Although the overall decline in the number of fatal falls overboard is encouraging, these largely preventable events remain a leading contributor to commercial fishing deaths. Implementation of prevention strategies discussed in this report by vessel owners could continue this positive trend and result in substantial safety improvements within the industry. Future research can include activities to understand barriers to adoption of these prevention strategies, particularly in fisheries where these events occur frequently, and evaluate the efficacy of interventions, as supported by the NIOSH strategic plan (*10*).

**Summary**

**What is already known about this topic?**

Commercial fishermen experience fatalities at a rate much higher than that of all U.S. workers, partially driven by falls overboard, a leading cause of work-related deaths in the industry.

**What is added by this report?**

During 2000–2016, 204 commercial fishermen died from unintentional falls overboard. Fifty-nine percent of falls were not witnessed, and 89.3% of these victims were not found. Among 83 witnessed falls, 22 victims were recovered but not resuscitated. None wore a personal flotation device (PFD).

**What are the implications for public health practice?**

Prevention strategies can be implemented to prevent future fatalities, including reducing fall hazards; using PFDs, man-overboard alarms, and recovery devices; and training crewmembers on resuscitation and treatment.

## Footnotes

* Fishery was defined as the fish species targeted and geographic location in which the fishing vessel was operating at the time of the event. Gear type was specified when multiple methods of harvesting could apply.

† A life ring is a circular flotation device carried on a vessel that can be used in a man-overboard recovery attempt. The life ring is attached to a line and can be thrown to the person in the water to provide immediate buoyancy and prevent drowning while rescue attempts continue.

§ The gunnel, also known as gunwale, is the uppermost edge of the side of a vessel.

¶ Requirements for Commercial Fishing Industry Vessels, 46 C.F.R., Chap. 1, Part 28; 2012.
