# *Erratum:* Vol. 67, No. 16

**DOI:** 10.15585/mmwr.mm6721a7

**Published:** 2018-06-01

In the report “Fatal Falls Overboard in Commercial Fishing — United States, 2000–2016” on page 469, in Figure 2, the number of falls “Witnessed, Recovery attempted within 1 hour, Recovery unsuccessful“ should have been **34** and not 5 as reported.

